# DXA reference values of the humanoid sheep model in preclinical studies

**DOI:** 10.7717/peerj.11183

**Published:** 2021-04-30

**Authors:** Christoph Biehl, Jakob Schmitt, Sabine Stoetzel, Deeksha Malhan, Fathi Hassan, Gero Knapp, Christian Heiss, Thaqif El Khassawna

**Affiliations:** 1 Department of Trauma-, Hand- and Reconstructive Surgery, Justus Liebig Universität Gießen, Giessen, Hesse, Germany; 2 Experimental Trauma Surgery, Justus Liebig Universität Gießen, Giessen, Hesse, Germany

**Keywords:** Large animal model, DXA, Bone mineral density (BMD), Bone mineral content (BMC), T-Score, PBM, Reference parameters, Osteoporosis

## Abstract

**Background:**

Merino land sheep are a popular pre-clinical large animal model in research on systemic skeletal diseases such as osteoporosis. Interpretation of studies is difficult because many reference parameters are missing or not established. This study aims to determine the reference parameters of the skeletal system (peak bone mass = PBM, T-Score). A defined standard allows an easier comparison of the study data of the animal model with human studies (T-Score).

**Materials and methods:**

A total of 116 Dual Energy X-ray Absorptiometry DXA measurements were performed on 74 untreated sheep. The average age of the animals was 57 months. The BMD, BMC, and fat content of the sheep were determined by the relevant human region of interest (ROI). From this, the PBM and from this the T-score for each of the animals were calculated.

**Results:**

Using 682 DXA measurements BMD and BMC were determined to provide an indication to PBM. For BMD a significant correlation to the age of the animals was observed (*p* = 0.043). A significant correlation was also seen for BMC (B) (*p* ≤ 0.001). In the age-dependent analysis, a widespread of values above the linear regression line was measured for both BMD and BMC between the 50th and 90th months of life. From an age of about 90 months, a wider spread of values below the linear regression line was found, although the average values continued to rise.

**Discussion:**

The evaluation of the 116 DXA measurements allowed the determination of the PBM for merino land sheep. With the help of the PBM, a T-score was calculated for each animal. The statistical analysis shows significant differences in BMD values between the different animal groups in each of the four ROIs investigated. Individual control or sham groups per study are therefore not sufficient. To improve comparability, an independent reference group should be established.

**Conclusion:**

An independent reference group for PBM and a T-score was established from four to six-year-old animals. The bone density increases with the age of the animals. Around the fourth year of life, a first peak could be observed. Also, after the seventh year of life, a further peak with the beginning plateau phase was observed. When compiling a group of animals for an osteoporosis model, animals from the age of seven years should, therefore, be used.

## Introduction

Merino land sheep have been established as a translational animal model for humans in the research of osteoporosis and new biomaterials in orthopedics and traumatology (Karlsson, Gerdhem & Ahlborg, 2005; Martini et al., 2001; Newman, Turner & Wark, 1995). Although animal models have their limitations, i.e sheep have a higher bone density than humans (Turner, 2001), some models are more suitable than others in specific fields of study (Arens et al., 2007; Liebschner, 2004). In orthopedic and trauma research, sheep model reflects similarities in body weight and size of bones to humans and osteointegration of implanted materials in bone tissue. Furthermore, the seasonal variation of about 5% in Marino Land Sheep (Thompson et al., 1995) is recapitulative to that of postmenopausal women (Gerdhem et al., 2004). Beside the handling advantage of sheep, the bone size and anatomy is most suitable for testing biomechanical and biological properties for innovative biomaterials in orthopedic and traumatology, which have to be examined in the original size using same the surgical technique planned for the clinical practice (Egermann, Goldhahn & Schneider, 2005; Reinwald & Burr, 2011). Whereas in patients, a clear standard defines healthy and diseased bone and allows the interpretation of biological mechanisms in response to biomaterials and implants, the baseline of bone density and quality remains unexplored in the sheep model. A suitable method for determining bone mineral density (BMD) and the resulting PBM is the Dual Energy X-ray Absorptiometry (DXA)(Blake & Fogelman, 2007; Kanis et al., 2013; Pouilles et al., 2000). The baseline for patients was set after measuring subjects of different ages, the PBM age was finally determined as 30 years old.

Therefore, establishing a standard and reference values (PBM) using the (DXA) as the gold standard for healthy sheep, will enhance the interpretation of values measured in studies in sheep with osteoporosis (Blake & Fogelman, 2007; Kanis et al., 2013; Pouilles et al., 2000).

This study aimed to establish osteodensitometry reference values for merino land sheep. The study hypothesized that the age-correlated BMD of merino land sheep correlates with human BMD.

## Material and Methods

The current study is a part of the trans-regional project (Special research area Transregio Collaborative research center (SFB/Transregio) 79—Materials for tissue regeneration in systemically diseased bone) aimed to establish osteoporotic bone status in a small and large animal model. Animal experiments were carried out according to the animal welfare act of the national institute of health and the guide for care and use of laboratory animals. This study was performed in full compliance with Institutional and German protection laws and approved by the ethical committee of the local governmental institution (“Regierungspraesidium Darmstadt”, permit no. Gen. No. V54-19 C 20/15 - F31/36 for T1; for T2: V54-19 C 20/15 –FU/1061; and for HA/biocomposit: No. V54-19 C 20/15 –FU/1032). All animal experiments were conducted at the central research facility of the Johann Wolfgang Goethe University in Frankfurt am Main.

**Figure 1:**
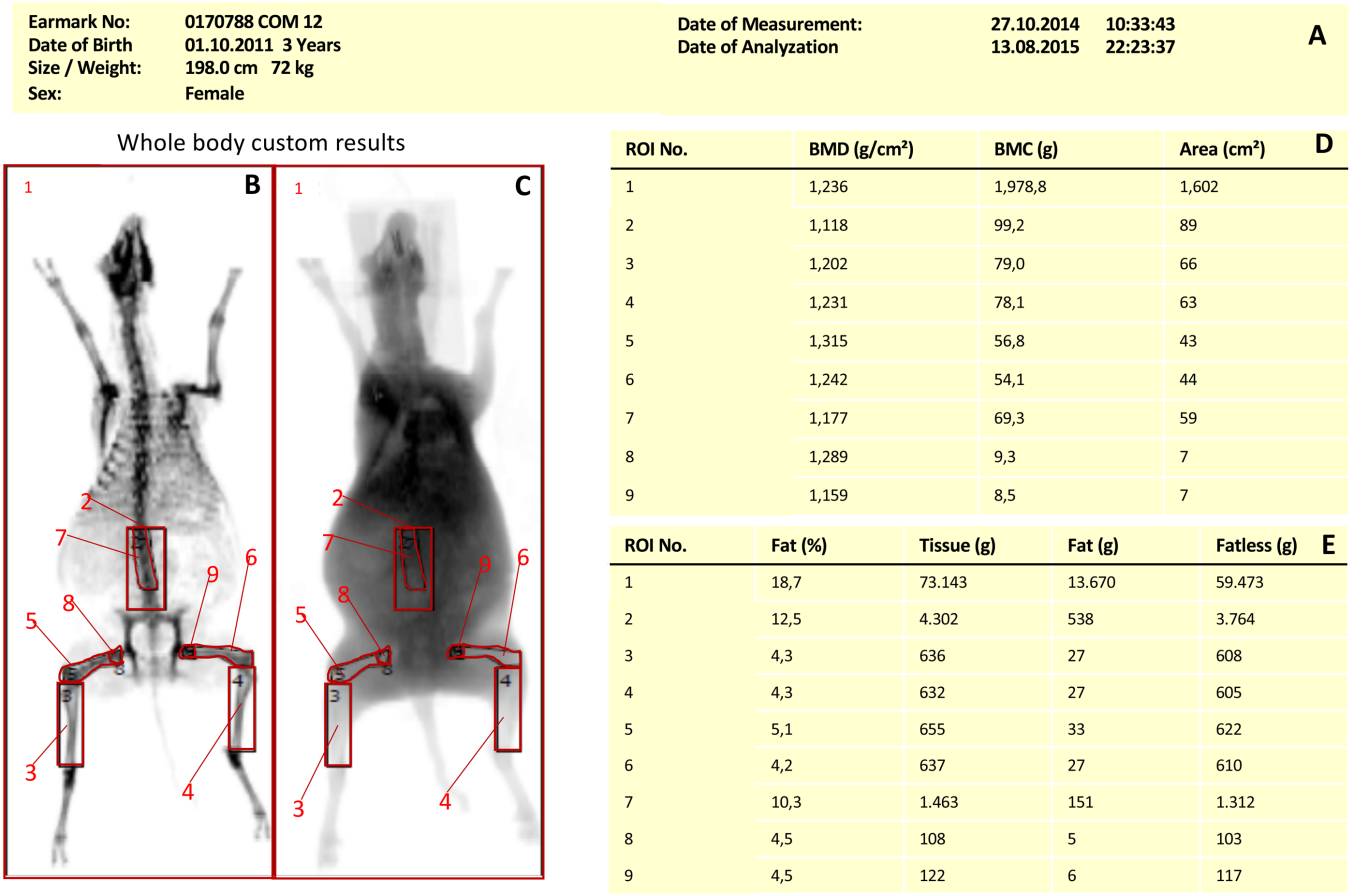
DXA Region of Interest in Merino land sheep. DXA scan Merino sheep with different regions of interest: Whole body (1), lumbar spine (2), left tibia (3), right tibia (4), left femur (5), right femur (6) upper lumbar spine (7), left femoral head (8), right femoral head (9). (A) General information about the test animal, (B) bone measurement, (C) soft tissue scan, (D) measurement results for the ROIs created in (B), (E) measurement results of the soft tissue window.

One hundred and sixteen DXA scans of 74 merino land sheep were analyzed. Group 1 (T1) and 2 (T2) included control animals from an osteoporosis research study. Group 3 (HA screw/biocomposite) was an animal model from an experimental study on the treatment of cruciate ligament rupture, sheep were otherwise healthy. Depending on the respective group, up to 9 regions of interest (ROIs) were evaluated in each measurement (Table 1). Figure 1 (DXA image) shows the different ROI selected for DXA measurement in sheep.

The animals were purchased from local breeders in Hesse and DXA scans were performed in collaboration with the University Hospital in Frankfurt am Main, where the animals were kept during the studies. The animals were pharmacologically untreated, not subjected to any specific diet and had sufficient exercise. The animals differed only in age, size, and weight. The average age of the animals was 57 (24–116) months. According to the relevance for human studies, BMD of the sheep was determined on the upper lumbar spine, both proximal femora and as a comparison of the individuals within the sheep flock on the whole body.

### Animal husbandry and feeding

The animals were kept under the supervision of experienced animal keepers and veterinarians, who guaranteed species-appropriate husbandry. The sheep were kept in their primary flock and allowed to free range on the premises of the Central Research Facility of Goethe University Frankfurt am Main, Germany. Water and food were regularly available. Since DXA measurement can only be performed on the resting patient/animal, a general anesthesia was required for the animals. Before the measurements, two animals were placed in a common stall box. In the last twelve hours before anesthesia, they received only water and no food. The premedication for the succeeding DXA procedure was also given in the boxes. Before each bone scan the weight of the animals was determined. The sheep average weight was 65.5 (41–111) kg.

### Experimental design

The current study investigated control groups (Sham animals) from two multicenter research projects (SFB TRR79) that aimed to study osteoporosis in sheep model. Therefore, the first group was named osteoporosis (T1) and the second osteoporosis (T2). However, no animal had any treatment that would induce osteoporotic bone status or other metabolic discrepancy in sheep bone. The third group utilized the data from a different project that aimed to investigate a novel interference screw for ACL reconstruction. Therefore, bilateral DXA scans were obtained before the first operation, and a unilateral scan was taken after 46 weeks. The animals from mentioned study are referred as (HA/Biocomposite) group. The DXA scans were performed on healthy Merino sheep, which can be considered representative of their species. No specific diet or surgery was conducted. In each study group scans were performed at the beginning of the study (0 months = 0M). Additionally, in group 1 (T1) after three and eight months (3M; 8M) and in group 3 (HA) after ten months (10M). In group 2 (T2) no additional scans were performed according to the study design (Table 2).

**Table 1:** Number and type of ROIs. Number and type of ROIs measured in each of the three animal groups.

**Group**	**measured ROIs**	**Regions of Interest (ROIs) with the corresponding identification numbers**
Group 1 Osteoporosis 1 (T1)	4	Whole body (1), upper lumbar spine (7), left femoral head (8), right femoral head (9)
Group 2 Osteoporosis 2 (T2)	4	Whole body (1), upper lumbar spine (7), left femoral head (8), right femoral head (9)
Group 3 ACL (Biocomposit + HA)	9	Whole body (1), lumbar spine (2), left tibia (3), right tibia (4), left femur (5), right femur (6) upper lumbar spine (7), left femoral head (8), right femoral head (9)

**Table 2:** Number and timing of bone density measurements. Overview of the number and timing of bone density measurements in the various study groups. Group 1 : 47 DXA in total. 31 measurements (0 M) and for 8 animals after 3- and 8- months standing time (3M, 8M). Group 2 : 15 measurements were taken at the beginning of the study (0M). Group 3 : 28 measurements were taken at baseline (0M) and 10 months later (10M).

	Number of bone density measurements (DXA)				
	0 M	3 M	8 M	10 M	Total
Group 1 Osteoporosis(T1)	31	8	8	–	47
Group 2 Osteoporosis(T2)	15	–	–	–	15
Group 3 ACL (Biocomposit + HA)	28	–	–	26	54
Total	74	8	8	26	116

In total, 116 scans were taken on 74 Merino sheep using the device Lunar Prodigy (GE Healthcare, Little Chalfont, United Kingdom). The scans were analyzed by enCORE Software (v13.40, GE Healthcare). Each day the device was calibrated using the enCORE Software and the manufacturer provided phantom bodies, which were used for calibration.

The animals were sedated by intramuscular application of 10 mg/kg Ketaminhydrochlorid (Ketavet^®^ 10 mg/ml, Bela- Pharm GmbH und Co.KG, Vechta), 0,01 ml/kg Xylazin (Rompun^®^ 2%, Bayer AG, Leverkusen), 0,3 mg/kg Midazolam (Midazolam Rotexmedica 5 mg/ml, Rotexmedica GmbH, Trittau) and 0,01 mg/kg Atropin (Atropinsulfat B. Braun 0,5 mg/ml, B. Braun Melsungen AG, Melsungen). Afterwards, the sedation was kept upright by intravenous application of Propofol (Propofol 2% (20 mg/ml), Fresenius Kabi Deutschland GmbH, Bad Homburg) using a perfusor (50 ml/h). The sedated animals were placed on the DXA-device according to the manufacturer‘s guidelines.

DXA scans focused on BMD, BMC as well as absolute fat content (fat (g)) and relative fat content (fat (%)) in 4 clinically relevant regions of interest (ROIs), namely whole body (ROI 1), upper lumbar spine (ROI 7), left and right femoral head (ROI 8 and 9).

### Independent reference group

For each merino land sheep, a T-score was calculated based on the BMD and the PBM values (Formula 1). Using Sheep from control groups from different studies enables researchers to obtain a baseline as a reliable alternative to the use of a new control group in future experiments. Such procedure is implemented in the clinical practice and if followed will reduce the number of animals required in new bone experiments. This study utilized scan measurements from animals between 48 and 72 months old to determine the baseline5 values.

Formula 1 Formula for calculating the T-score in Merino Land Sheep

T − Score= $ \frac{ \left( \text{measured BMD}-\text{mean value of the BMD of all animals at the age of}48-72\text{month} \right) }{\text{standard deviation of the mean BMD of the animals at the age of}48-72\text{month}} $

### Statistical analysis

**Figure 2:**
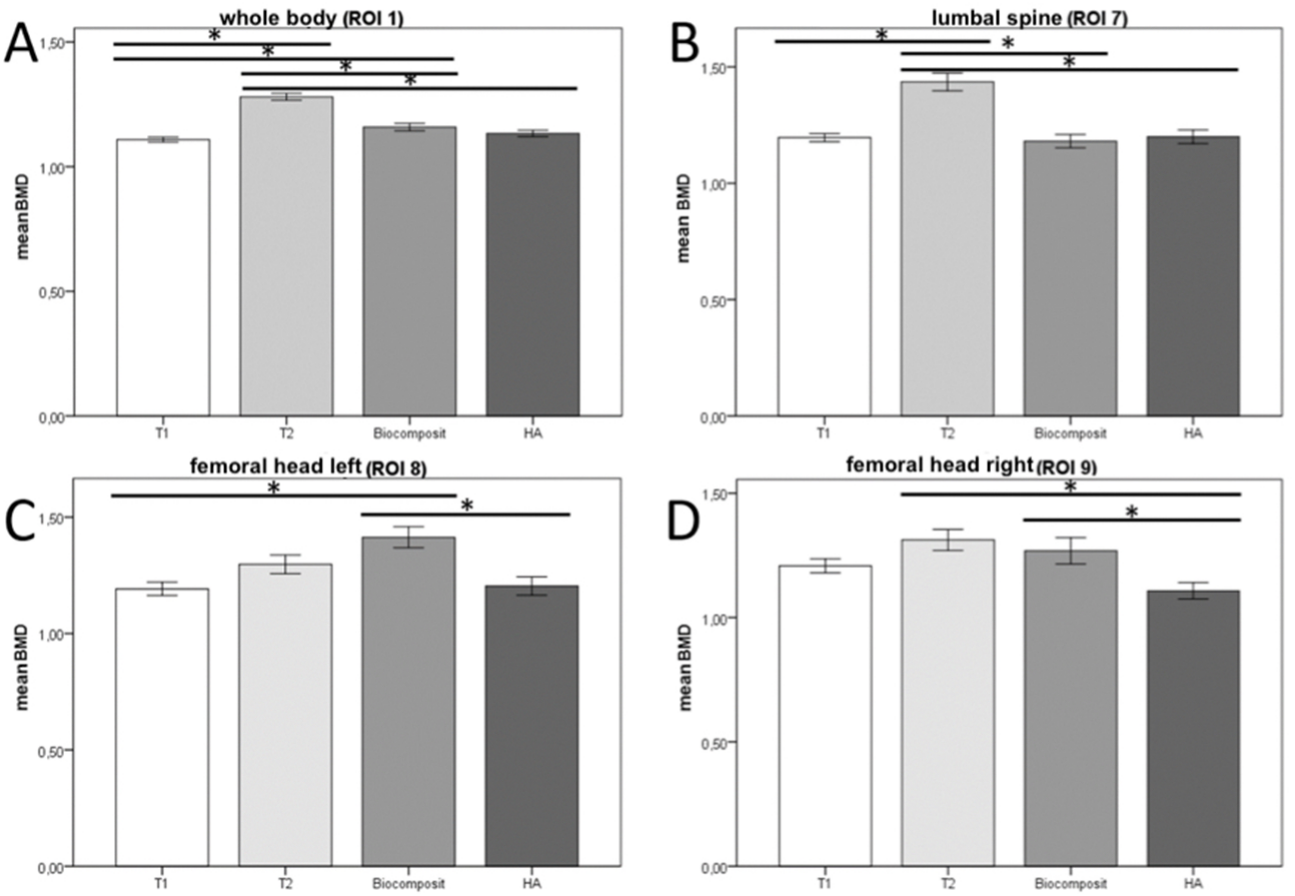
Mean BMD in different ROIs. With the Bonferroni correction, the BMD values of the individual test groups were tested for significant differences. The figure shows the mean BMD values of the ROIs whole body (A), upper lumbar spine (B), left (C) and right (D) femoral heads. * = Significant difference. The Biocomposite and HA groups together form Group 3 but are listed as two separate groups in this graph. Level of significance: *p* ≤ 0.05. Analysis in SPSS.

The statistical evaluation of the bone scans was performed descriptively and quantitatively by using the software SPSS (V. 24.0, IBM, CA, ). The measured values of the parameters of BMD, BMC, age, weight, fat (%) and fat (g) were evaluated. The analysis focused on the correlation between age and BMD of the animals, PBM was calculated out of the measured BMDs of all scans. Data were examined for normal distribution and was found non-parametric. Therefore, Mann–Whitney *U* test was used to examine the significance considering a cutoff of *p* ≤ 0.05. Bar graphs were plotted as Mean ± Standard Error of Mean (Mean ± SEM) (Fig. 2).

**Figure 3:**
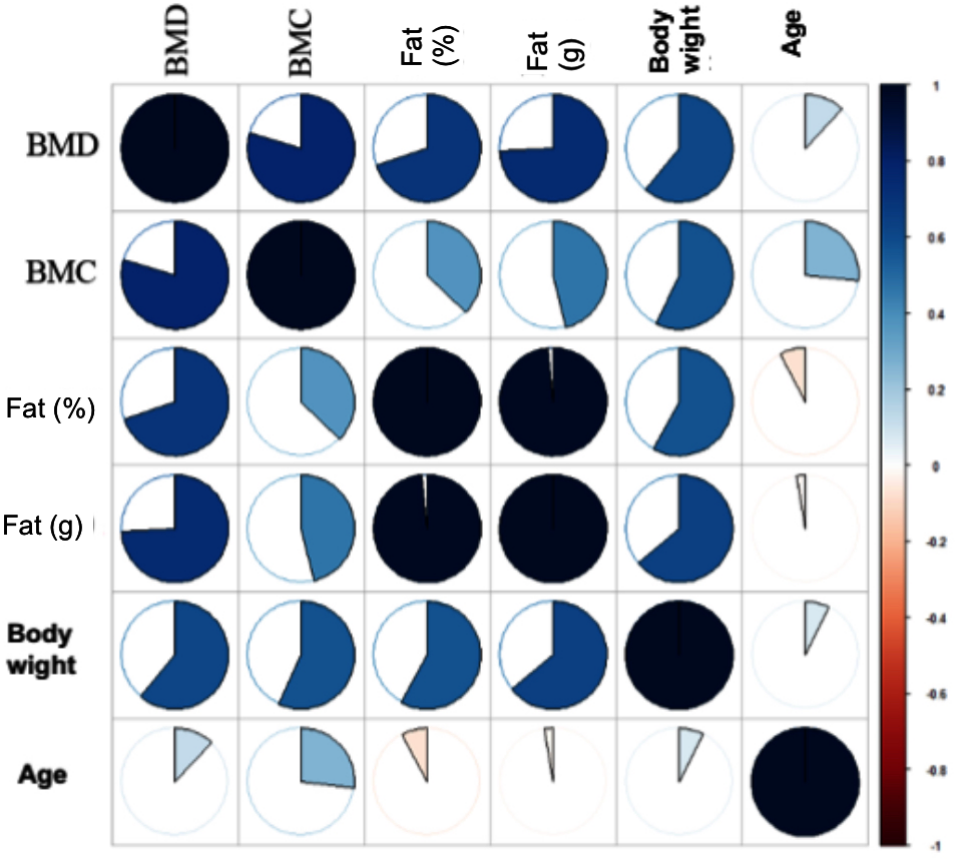
Heatmap for correlation coefficient for ROI whole body. The heatmap gives an overview of the correlation coefficient according to Spearman for the ROI whole bodies. The darker the coloration of a diagram, the higher the correlation coefficient of the two parameters (maximum value 1). In addition, the extent of the correlation can be seen from the pie charts shown. There are significant correlations between BMD and BMC, weight, fat (%) and fat (g). Level of significance: *p* ≤ 0.05. Analysis in R.

**Figure 4:**
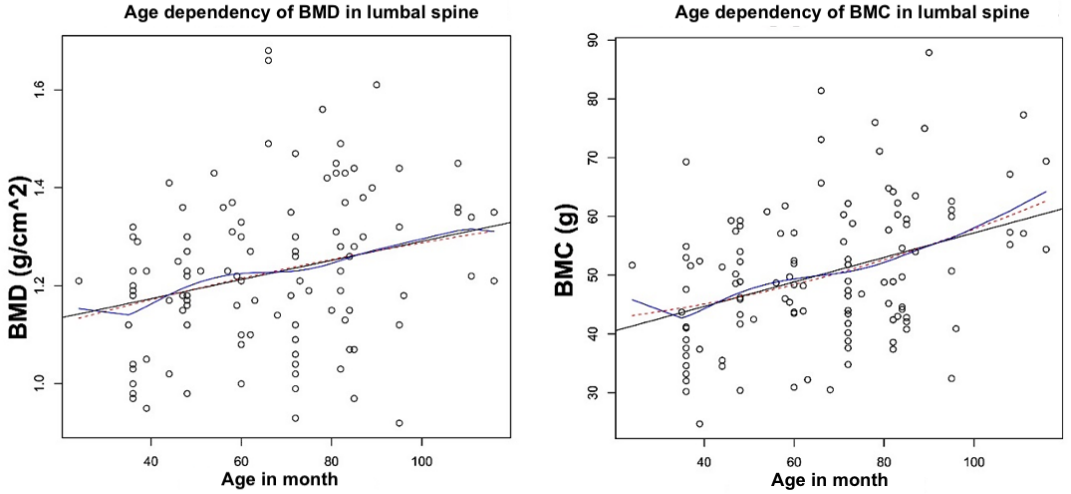
Age-dependent correlation of BMD and BMC in ROI 7. Age-dependent course of BMD and BMC in ROI7, upper lumbar spine. (A) For BMD a significant correlation to the age of the animals could be demonstrated (*p* = 0.043). (B) When considering the BMC, a significant correlation was also observed (*p* ≤ 0, . 001). The correlation coefficient was calculated using the Pearson method at *p* ≤ 0, . 05. Black: linear regression line, blue: quadratic regression curve, red: non-parametric regression. Analysis in R.

Further analysis were carried out using the statistical software R (The Comprehensive R Archive Network (https://cran.r-project.org/)). The measured values were examined in relation to the various ROIs and study groups. Using the ‘ggplot2’ package built in R, scatter plots were created (Yavorska & Burgess, 2017). Furthermore, the corrplot package in R (https://cran.r-project.org/web/packages/corrplot/index.html) was used to display the correlation matrix in the form of a heat map (Fig. 3). Using the core Package in R (Cores of Recurrent Events) (Yavorska & Burgess, 2017) (Version 3.4.0, R Core Team, 2018) to assess regression line, quadratic regression, as well as non-parametric regression and the correlation coefficient according to Spearman’s rank correlation coefficient (Figs. 4A and 4B).

## Results

The PBM was determined by evaluating all bone density scans, based on which a T-score was calculated. A correlation between the increasing age of the animals and a decrease in bone density or the development of osteopenia or osteoporosis was shown. Analogous to the clinical routine, special attention was paid to the bone density of the ROIs upper lumbar spine (7) as well as left (8) and right femoral head (9).

### Descriptive statistics

BMD represents bone mineralization and is the most important value of osteodensitometry. In 682 BMD measurements, the average BMD was 1.2 (0.79–2.09) g/cm^2^ (Table 3).

BMC as an area-independent value is another important parameter in osteodensitometry. For 682 measurements, the mean BMC value was 341.81 (4.1–2973.2) g.

In addition, the relative and absolute fat content within the respective ROI was determined from the DXA scans. Fat is found both in the soft tissue surrounding the bone and in the bone marrow. The values were used to check whether a high fat content has an influence on the measured bone density and is associated with the development of osteoporosis. The mean value of the relative fat content was 6.94 (4–29) % based on 673 measurements (Table 3). For the absolute fat percentage, based on 672 measurements, the mean value was 1613.35 (2–31978) g.

### BMD measurements in different ROIs

The BMD values were considered separately for each ROI.

In ROI 1 (whole body), the BMD values averaged 1.15 (0.98–1.38) g/cm^2^ (minimum-maximum) (Table 4). The values showed a normal distribution *(skewness: 0.27; kurtosis: −0.35)*.

**Table 3:** Descriptive statistics of all collected parameters. For each bone density measurement, the six parameters weight, age, BMD, BMC, relative, and absolute fat content were collected. The table provides an overview of the minima and maxima of the values, as well as the mean value and standard deviation.

	Number of measure-ments	Minimum	Maximum	mean		standard deviation
		Statistics	Statistics	Statistics	Std. error	Statistics
Weight (kg)	716	41	111	65.5056	0.48677	13.02507
Age (Month)	734	24	116	66.2125	0.75775	20.52918
BMD (g/cm^2^)	682	0.79	2.09	1.1975	0.00652	0.17022
BMC (g)	682	4.1	2973.2	341.813	25.83268	674.6239
Tissue (%Fat)	673	4	29.6	6.9374	0.19348	5.01918
Fat (g)	672	2	31978	1613.351	172.49227	4471.51077
Valid values	656					

**Table 4:** BMD values in DXA-ROI of merino sheep. Overview of the BMD values measured in ROIs 1, 7, 8 and 9. Minimum, maximum, mean, and standard deviation were determined from 116 measurements. Kurtosis and skewness (not listed in the table) were used to check the parameters for normal distribution.

		Number of measurements	Minimum	Maximum	Mean	Std-deviation
Whole body (1)	BMD (g/cm^2^)	116	0.98	1.38	1.15	0. 09
Upper lumbar spine (7)	BMD (g/cm^2^)	116	0.92	1.68	1.22	0.16
Left femoral head (8)	BMD (g/cm^2^)	116	0.87	2	1.26	0.22
Right femoral head (9)	BMD (g/cm^2^)	116	0.79	2.09	1.21	0.22

In ROI 7 (upper lumbar spine), the mean value was 1.2 g/cm^2^. BMD values ranged between 0.92 g/cm^2^ (minimum) and 1.68 g/cm^2^ (maximum). The measured values were normally distributed (skewness: 0.37; kurtosis: −0.49).

For ROI 8 (left femoral head) the mean value was 1.26 (0.87–2.0) g/cm^2^. The values were normally distributed (skewness: 0.55; kurtosis: 0.19) (Table 4).

Evaluation of BMD in ROI 9 (right femoral head) yielded values between 0.79 g/cm^2^ (minimum) and 2.09 g/cm^2^ (maximum). The mean value was 1.21 g/cm^2^. The values were not normally distributed (skewness: 0.95; kurtosis: 1.62).

### Differences between the experimental groups

The BMD values of the measured ROIs (1,7,8,9) were collected and compared separately for each group. In group 3 animals, HA and biocomposite were analyzed separately. Bonferroni correction was applied as a post-hoc procedure (Fig. 2), with which the BMD values of the experimental groups were checked for significant differences (*p*-value ≤ 0.05).

In ROI 1 (whole body) (Fig. 2A) BMD values differed significantly between the groups T1 and T2 {*p* < 0.001} and T1 and biocomposite {*p* = 0.016}. Also, between group T2 and biocomposite {*p* < 0.001}, T2 and HA {*p* < 0.001}.

In ROI 7 (upper lumbar spine) (Fig. 2B) the analysis showed significant differences between groups T1 and T2 {*p* < 0.001}, T2 and biocomposite {*p* < 0.001} and T2 and HA {*p* < 0.001}.

In ROI 8 (left femoral head) (Fig. 2C) the BMD values showed only significant differences between the values of groups T1 and biocomposite {*p* < 0.001}, and groups biocomposite and HA {*p* = 0.001}.

Statistical analysis of ROI 9 (right femoral head) (Fig. 2D) revealed significant differences between the results of groups T2 and HA {*p* = 0.016} and biocomposite and HA {*p* = 0.031}.

### Correlations between BMD and other bone density parameters

Bone density cannot be considered independently of the other measured parameters. Spearman rank correlation coefficient was determined to assess the relationship between BMD and the parameters weight, age, BMC and fat. This was initially recorded independently of the animal groups for the region’s whole body, upper lumbar spine, and left and right femoral head (Table 5 and Heatmap: Fig. 3). The significance level was *p* ≤ 0.05. It was then also collected separately for each group.

**Table 5:** T-score of merino sheep. BMD measurements used to calculate the T-score of merino sheep aged between 48 and 72 months. For each of the four relevant regions, 48 measurements could be used.

		Whole body	Upper lumbar spine	Left femoral head	Right femoral head
Measurements		48	48	48	48
Mean		1.13	1.21	1.25	1.21
Median		1.12	1.2	1.22	1.17
Standard deviation		0.08	0.16	0.22	0.24
Minimum		0.98	0.93	0.87	0.79
Maximum		1.37	1.68	2.00	2.09

In ROI 1: whole body, (Table 6; Fig. 2) significant correlation between BMD and body weight was observed (*p* ≤ 0.001). The correlation between BMD and BMC was significant and also for BMD with the absolute and relative fat content (*p* ≤ 0.001; *p* ≤ 0.001). However, the correlation of BMC and BMD with age were not significant. In the upper lumbar spine area, significant correlations were found between BMD and all other parameters surveyed (Table 6).

**Table 6:** BMD-Spearman rank correlation. Spearman rank correlation of the BMD to the parameters weight, age, BMC, relative fat percentage (fat %) and absolute fat percentage (fat (g)). The correlations were determined for each of the four regions (whole body, upper lumbar spine, left and right femoral head). The significance level was *p* < 0.05.

		weight (kg)	Age (Month)	BMD (g/cm^2^)	BMC (g)	Tissue (%Fat)	Fat (g)
BMD whole body	Correlation coefficient	0.61^∗∗^	0.11	1.000	0.79^∗∗^	0.70^∗∗^	0.74^∗∗^
	significance (2-side)	≤0.001	0.24		≤0.001	≤0.001	≤0.001
BMD upper lumbar spine	Correlation coefficient	0.37^∗∗^	0.26^∗∗^	1.000	0.79^∗∗^	0.55^∗∗^	0.51^∗∗^
	significance (2-side)	≤0.001	0.01		≤0.001	≤0.001	≤0.001
BMD left femoral head	Correlation coefficient	0.15	0.05	1.000	0.43^∗∗^	0.07	−0.02
	significance (2-side)	0.12	0.61		≤0.001	0.50	0.82
BMD right femoral head	Correlation coefficient	0.15	0.15	1.000	0.45^∗∗^	0.15	−0.09
	significance (2-side)	0.11	0.12		≤0.001	0.11	0.36

The evaluation of the ROI of the left femoral head showed a significant correlation between BMD and BMC values (Table 6). The correlations between BMD and the other measured values were not significant.

Also, in the ROI of the right femoral head, a significant correlation was seen between BMD and BMC (Table 6).

To calculate the T-score, a specific reference group was formed (see Table 5). For each of the four relevant ROIs, 48 measurements of animals aged between 48 and 72 months could be evaluated.

### Regression models for the age-dependent course of bone density parameters

Using regression models, the age-dependent course of BMD and BMC was examined. For BMD, the linear regression model showed a significant steady increase in ROI 7, upper lumbar spine *p* = 0.04. For BMC, significant increases were found in the linear regression models of regions 1 (whole body), 7 (upper lumbar spine) and 8 (left femoral head). In all regions, there was a wide range of values (Table 7).

**Table 7:** Regression models. Overview of the regression models for the relationship between age and BMD or BMC in the various ROIs. Linear, quadratic and nonparametric regression were considered. The linear regression line shows significant slope values for the BMD in ROI 7, for the BMC this is found in ROI 1, ROI 7 and ROI 8. There are no significant values for the quadratic regression. The coefficient of determination R indicates widely scattered values in all regions. Level of significance: *p* ≤ 0, 05.

	**Linear model**		**Quadratic model**			**Nonparametric regression**
**Characteristic**	**Significance progression (*p*-value)**	**R**^**2**^	**Significance b1 (*p*-value)**	**Significance b2 (*p*-value)**	**R**^**2**^	**R^2^**
BMD ROI 1	0.14	0.019	0.83	0.63	0.021	0.028
BMC ROI 1	≤0.003	0.076	0.081	0.21	0.089	0.0997
BMD ROI 7	≤0.004	0.069	0.48	0.84	0.0696	0.084
BMC ROI 7	≤0.001	0.14	0.86	0.56	0.14	0.16
BMD ROI 8	0.71	0.001	0.12	0.13	0.022	0.055
BMC ROI 8	0.017	0.049	0.06	0.14	0.068	0.085
BMD ROI 9	0.13	0.02	0.91	0.88	0.021	0.041
BMC ROI 9	0.068	0.029	0.25	0.4	0.035	0.046

In the quadratic regression model, no significance was found in the determined regression curves for either BMD or BMC. Here, too, there was a widespread value. For BMD, the values ranged between (*R*^2^ = 0.021) in ROI 1 and ROI 9 and (*R*^2^ = 0.0696) in ROI 7. The BMC yielded coefficients of determination between (*R*^2^ = 0.035) in ROI 9 and (*R*^2^ = 0.14) in ROI 7.

### Exemplary consideration of the course of BMD and BMC in ROI 7, upper lumbar spine

Due to higher incidence of osteoporotic fracture in lumbar vertebrae, the course of BMD and BMC in ROI 7: upper lumbar spine was seen, as an example (Fig. 4).

In the age-dependent view of BMD (Fig. 4A), a significant correlation was found in the Pearson method. Higher BMD values are more frequently observed from the age of about 50 months, while the dispersion of values above the linear regression line increases up to the age of 90 months. The maximum value is observed between 60 and 80 months of age. From an age of about 90 months, a wider dispersion of BMD values below the linear regression line is observed, although the average BMD value continues to rise.

The age-dependent pattern of the BMC also shows a significant correlation of the slope of the linear regression line in the Pearson method (Fig. 4B). From the 40th month onwards, the dispersion of values above the linear regression line increases. The maximum value is reached at 90 months. After that, a wider dispersion below the linear regression line is observed, while the average BMC value continues to rise, similar to the BMD.

The distribution of the quadratic regression curves of BMD and BMC was not significant.

## Discussion

The aim of the study is to determine osteodensitometry reference values for merino land sheep in order to enable the transferability of a preclinical study in animal models to humans. The human T-score reference values are based on data from the NHANES III study (Hanson, 1997; Lewiecki et al., 2008; Looker et al., 1997).

The evaluation of 116 DXA measurements of 74 animals from projects of a Collaborative Research Centre enabled the determination of the PBM for merino land sheep. Further parameters such as BMD and BMC, which were also determined, facilitate the interpretation of the results with comparable studies and with human measurements (Heaney et al., 2000). With the help of PBM, T-score could be calculated for each animal. According to the literature, merino land sheep will reach skeletal maturity at the age of three to four years (Augat et al., 2003; Cake et al., 2006; Pearce et al., 2007; Reinwald & Burr, 2011).

### Control groups in current large animal studies

Currently, studies using large animal models often do not use independent reference values. The implementation of the ARRIVE guidelines has contributed to a standardized documentation and publication of results from animal models (Kilkenny et al., 2010). Initial values must be redefined and/or an untreated control group must be used at the beginning of each study (Zarrinkalam et al., 2009). By applying the data from this study, the number of sheep used in animal models can be reduced by up to 50% (reduction). Nevertheless, the goal remains a complete replacement of animal experiments. In addition, the establishment of reference values can significantly improve the meaningfulness and transferability of results through a more targeted selection of test animals.

In the present study, significant differences in the BMD of the individual groups in each of the four ROIs were observed (Fig. 2). We conclude that individual control or sham groups per study are not sufficient. The large animal model of sheep does not represent a uniform genetic population, therefore the variation in values of the animals is greater than in rodents. To improve the comparability between the results of different bone density studies, it is necessary to establish an independent reference group.

### Factors influencing BMD

In earlier studies a connection between BMD, osteoporosis and increased fat content in the bone could be observed (Heiss et al., 2017; Rosen & Bouxsein, 2006; Sekiya et al., 2004). Age also influences the bone density of the animals (Reinwald & Burr, 2011).

In DXA scans, therefore, in addition to BMD and BMC, the relative and absolute fat content in the ROI investigated was also measured (Miller et al., 2018).

As with BMD, significant differences were found between the four ROI compared to the other parameters. The large interindividual differences between the animals could not provide comparable results, so this should be regarded as further evidence of the need to form a reference group.

### The influence of age on bone density parameters

The physiological course of bone metabolism in merino land sheep is still largely unexplored. In the literature, the age of three to four years is given for the sheep to reach skeletal maturity. The BMD and BMC values measured in this study were age correlated to determine the time of PBM and its further course. Our data show the first peak around the age of four years. Unlike humans, bone density does not remain on a plateau for several years after this first peak but increases steadily with age in sheep. Around the seventh year of life a further peak can be seen, followed by a plateau phase.

### Influence on the design of future studies

Osteoporosis is usually a disease of old age (EPOS et al., 2002), which occurs decades after patients have reached skeletal maturity. Therefore, a suitable animal model should also have already reached skeletal maturity in order to allow a better transferability of the results to humans. The data of this study on the age-dependent course of the disease suggest that merino land sheep reach a plateau phase of bone density around the age of seven. In order to avoid unnecessary experiments on unsuitable animals, animals aged seven years or older should therefore be used in future studies on osteoporosis in merino land sheep. In addition to the four ROIs investigated here, the publication of the raw data from this study will allow reference values to be established for each bone region of the animals. For example, sheep are often used in neurosurgical preclinical research as a model of the human cervical spine (Long et al., 2018). Independent reference values of bone density in the cervical spine region could be obtained quickly and easily using our raw data.

For this study, female bone-healthy merino land sheep were examined without exception. The data are also made publicly available as open-source documents at http://DXAdb.glycosciences.de/. A database with reference values for male animals and/or animals with osteoporosis would simplify the planning of future studies, reduce possible sources of error and thus lead to more effective use of the animal models.

In terms of refinement, clinical studies can be planned more effectively, using animals who represent the bone characteristics of the mainly affected age group in humans.

Future studies can benefit from the results by choosing animals in specific age groups according to their study aims. Furthermore, the number of animals in studies can be reduced by sparing reference groups in osteoporotic studies according to 3R (Hooijmans, Leenaars & Ritskes-Hoitinga, 2010; Russell & Burch, 1959).

## Conclusion

With regard to the 3Rs, reduction, refinement and replacement, this study aimed to establish reference values for bone density in merino land sheep. On the basis of these values, a reference group of the species was to be formed analogous to the T-score in humans. A corresponding group could finally be formed from 48 measurements of animals aged between four and six years. With the age of the animals a steady increase in bone density was observed. Unlike the human being, two peaks observations are made with the merino sheep. The first around the fourth year of life and the second from around the seventh year of life. Only after the second peak does a plateau phase follow, as in humans. According to our data, merino sheep from the age of seven years should be used when assembling a group of animals for an osteoporosis model. A more targeted selection of the experimental animals can thus significantly improve the clinical transferability of the study results. Hereby the number of sheep required in the osteoporosis model can be significantly reduced.

##  Supplemental Information

10.7717/peerj.11183/supp-1 Supplemental Information 1 Calibration protocol for DXA The calibriationprotocol guides as a step-by-step guide to DXA measurement in sheep.

10.7717/peerj.11183/supp-2 Supplemental Information 2 Group distribution of the experimental sheep Group distribution of the experimental sheep with corresponding ear tag number, weight in kg, age at the beginning of the experiment as well as the median age of the corresponding groups in years.

10.7717/peerj.11183/supp-3 Supplemental Information 3 Raw data Raw data from sheep testing at the laboratory.

## Abbreviations

3R: Ethical principle of the “3Rs”: Replace, Reduce, Refine
ACL: anterior cruciate ligament
ARRIVE: A Randomized Trial of Induction Versus Expectant Management
BMC: bone mineral content
BMD: bone mineral density
DFG: Deutsche Forschungsgemeinschaft
DXA: Dual Energy X-ray Absorptiometry
HA: hydroxyl apatite
NHANES III: Third National Health and Nutrition Examination Survey
PBM: peak bone mass
ROI: region of interest
SPSS: Statistical Package for the Social Sciences
SFB: Sonder-Forschungs-Bereich
TR: Trans Regio
T-score: Osteoporosis value in g/cm2
ZFE: Central Research Facility

## Additional Information and Declarations

### Data Availability

The following information was supplied regarding data availability: Raw data are available in the Supplemental Files.
