# Supplementary material for: DXA reference values of the humanoid sheep model in preclinical studies
Source: PeerJ. 2021 Apr 30;9:e11183. doi: 10.7717/peerj.11183 (PMC8092102; doi:10.7717/peerj.11183)
Supplement: Supplemental Information 1 — The calibriationprotocol guides as a step-by-step guide to DXA measurement in sheep. [file peerj-09-11183-s001.docx]

**Calibration protocol**

The DXA device was prepared for its use before the test started. After switching on the device and the connected computer, calibration and quality control were performed daily. The procedures were predetermined by the software used and were always performed in the same way.

The button Quality Control (F5) in the start menu of the software leads to the corresponding menu (Figure 1). At first, an overview of the last quality controls is shown. The traffic light symbol shows the current system status. If the device has not been calibrated for 48 hours, the traffic light is red (Figure 2). No scans can be made. New quality control and calibration must be performed to obtain the software release.

Two reference bodies were used to calibrate the instrument (Figure 3A). These are placed in fixed positions on the patient couch of the DXA device and fixed (Figure 3B). The so-called phantom body is designed to simulate the density of the vertebral bodies. During the calibration process, a container filled with water is placed on top of the phantom body to simulate the soft tissue.

The entire process was controlled by the enCORE software. A sequence of different tests was performed (Figure 4). Only when all tests had been successfully completed was the release to perform the bone scans given.


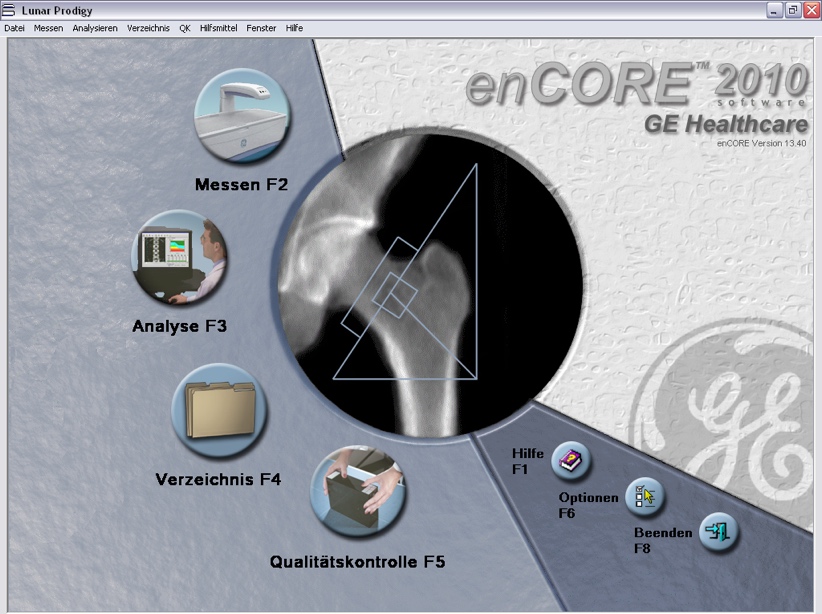


Figure 1: Start screen of the enCORE software. By clicking on the Quality Control icon (F5) in the start screen, the user gets to the corresponding menu (Figure 2).


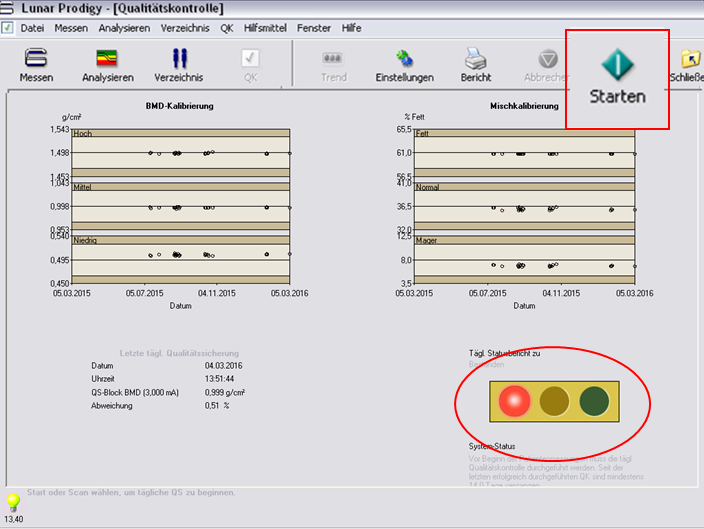


**Figure 2:** Overview of the last quality controls. The system traffic light is set to red (red circle), without performing a calibration, the software will stop DXA measurements. The calibration can be started by clicking on the Start icon.


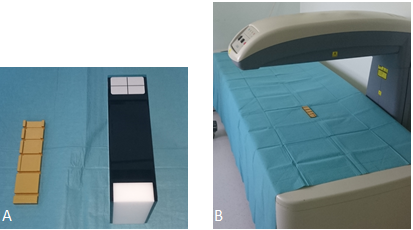


Figure 3: (A): The two phantom test bodies used to calibrate the DXA device, which simulates the density of human vertebral bodies. On the right, the vessel for water, which simulates soft tissue. (B): The phantom body has already been placed on the couch. To simulate the soft tissue, the container with water is placed on top of it.


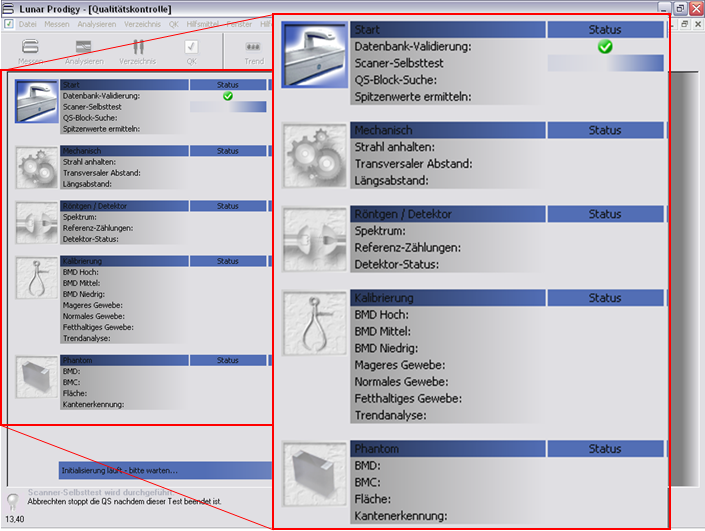


**Figure 4:** Software protocol (enCORE Software; v13.40, GE Healthcare) during the calibration process. The progress and results of the tests are displayed. When the status is green, the device is calibrated, and the measurements can be performed.
