# Supplementary material for: DXA reference values of the humanoid sheep model in preclinical studies
Source: PeerJ. 2021 Apr 30;9:e11183. doi: 10.7717/peerj.11183 (PMC8092102; doi:10.7717/peerj.11183)
Supplement: Supplemental Information 2 — Group distribution of the experimental sheep with corresponding ear tag number, weight in kg, age at the beginning of the experiment as well as the median age of the corresponding groups in years. [file peerj-09-11183-s002.docx]

| Group | number (intern) | Earmark ID | Age to  start of experiment in years | median age | weight in kg |
| --- | --- | --- | --- | --- | --- |
| Control | K1 | 68489 | 3 | 4,5 | 58 |
| Control | K2 | 83207 | 3 |  | 53 |
| Control | K3 | 83193 | 4 |  | 54 |
| Control | K4 | 55500 | 5 |  | 52 |
| Control | K5 | 94902 | 3 |  | 81 |
| Control | K6 | 83210 | 6 |  | 61 |
| Control | K7 | 48689 | 7 |  | 77 |
| Control | K8 | 32 | 9 |  | 70 |
| OVX | O1 | 68484 | 3 | 5 | 56 |
| OVX | O2 | 83205 | 3 |  | 53 |
| OVX | O3 | 83206 | 4 |  | 56 |
| OVX | O4 | 83211 | 5 |  | 60 |
| OVX | O5 | 83188 | 6 |  | 58 |
| OVX | O6 | 30 | 9 |  | 74 |
| OVX | O7 | 5 | 6 |  | 81 |
| OVXD | OD1 | 68483 | 3 | 4,5 | 47 |
| OVXD | OD2 | 83186 | 8 |  | 55 |
| OVXD | OD3 | 68488 | 4 |  | 54 |
| OVXD | OD4 | 83214 | 4 |  | 65 |
| OVXD | OD5 | 83209 | 5 |  | 59 |
| OVXD | OD6 | 94901 | 3 |  | 86 |
| OVXD | OD7 | 3 | 7 |  | 79 |
| OVXD | OD8 | 68473 | 7 |  | 56 |
| OVXDC | ODC1 | 83199 | 3 | 5,5 | 61 |
| OVXDC | ODC2 | 83212 | 3 |  | 41 |
| OVXDC | ODC3 | 83202 | 4 |  | 61 |
| OVXDC | ODC4 | 83190 | 5 |  | 67 |
| OVXDC | ODC5 | 83201 | 6 |  | 65 |
| OVXDC | ODC6 | 6 | 7 |  | 60 |
| OVXDC | ODC7 | 46 | 9 |  | 60 |
| OVXDC | ODC8 | 83195 | 6 |  | 53 |

Supplementary Material/Table:

Group distribution of the experimental sheep with corresponding ear tag number, weight in kg, age at the beginning of the experiment as well as the median age of the corresponding groups in years.


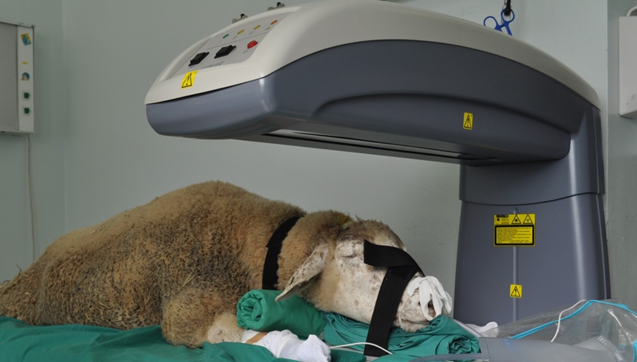


The sheep is fixed on the lying surface of the apparatus for examination by DXA.


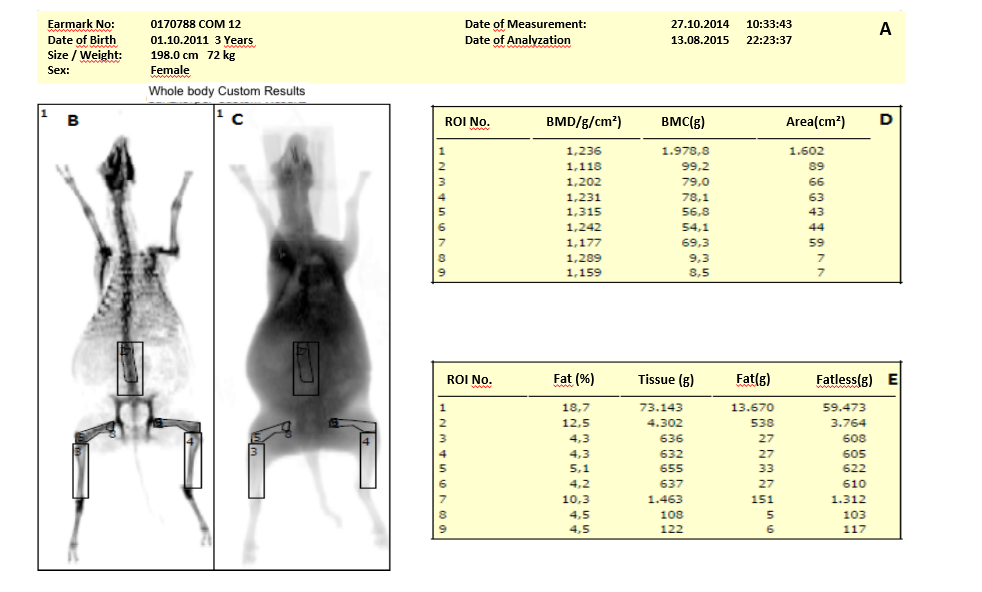


DXA analysis sheet
